# RETRACTION: MicroRNA‐320a Inhibits Invasion and Metastasis in Osteosarcoma by Targeting Cytoplasmic Polyadenylation Element‐Binding Protein 1

**DOI:** 10.1002/cam4.71140

**Published:** 2025-08-18

## Abstract

**RETRACTION:** WangY. , YangJ. , ChenP. , SongY. , AnW. , ZhangH. , ButegeleqiB. , and YanJ. , “MicroRNA‐320a Inhibits Invasion and Metastasis in Osteosarcoma by Targeting Cytoplasmic Polyadenylation Element‐Binding Protein 1,” Cancer Medicine 9, no. 8 (2020): 2833–2845, 10.1002/cam4.2919.32064777 PMC7163091

The above article, published online on 17 February 2020 in Wiley Online Library (http://onlinelibrary.wiley.com/), has been retracted by agreement between the journal Editor‐in‐Chief, Stephen Tait; and John Wiley & Sons Ltd. A third party reported that tumor images in Figures 2C and 6A had been duplicated from Figures 2C and 6A in a previously published article by different authors which involved different experimental conditions [Zou et al. 2016 (https://doi.org/10.18632/oncotarget.5689)]. The authors responded to an inquiry by the publisher and explained that the tumor images had been downloaded from the other article as a morphological reference and inadvertently were re‐used during the figure preparation stage for their own article. The authors supplied what they described as the correct tumor images, but they did not supply all original data as requested by the publisher. Without all of the original data, the new images could not be validated. The retraction has been agreed to because the unattributed duplication of images from an earlier publication, which describes different experimental conditions, fundamentally compromises the editors’ confidence in the results presented in this article. The authors did not respond to our notice regarding the retraction.

**RETRACTION:** Y. Wang , J. Yang , P. Chen , Y. Song , W. An , H. Zhang , B. Butegeleqi , and J. Yan , “MicroRNA‐320a Inhibits Invasion and Metastasis in Osteosarcoma by Targeting Cytoplasmic Polyadenylation Element‐Binding Protein 1,” Cancer Medicine 9, no. 8 (2020): 2833–2845, 10.1002/cam4.2919.32064777 PMC7163091

The above article, published online on 17 February 2020 in Wiley Online Library (http://onlinelibrary.wiley.com/), has been retracted by agreement between the journal Editor‐in‐Chief, Stephen Tait; and John Wiley & Sons Ltd. A third party reported that tumor images in Figures 2C and 6A had been duplicated from Figures 2C and 6A in a previously published article by different authors which involved different experimental conditions [Zou et al. 2016 (https://doi.org/10.18632/oncotarget.5689)]. The authors responded to an inquiry by the publisher and explained that the tumor images had been downloaded from the other article as a morphological reference and inadvertently were re‐used during the figure preparation stage for their own article. The authors supplied what they described as the correct tumor images, but they did not supply all original data as requested by the publisher. Without all of the original data, the new images could not be validated. The retraction has been agreed to because the unattributed duplication of images from an earlier publication, which describes different experimental conditions, fundamentally compromises the editors’ confidence in the results presented in this article. The authors did not respond to our notice regarding the retraction.

